# Expression and (Lacking) Internalization of the Cell Surface Receptors of *Clostridioides difficile* Toxin B

**DOI:** 10.3389/fmicb.2018.01483

**Published:** 2018-07-04

**Authors:** Dennis Schöttelndreier, Katrin Seeger, Guntram A. Grassl, Markus R. Winny, Robert Lindner, Harald Genth

**Affiliations:** ^1^Institute for Toxicology, Hannover Medical School, Hanover, Germany; ^2^Institute of Medical Microbiology and Hospital Epidemiology and DZIF Partner Site Hannover-Braunschweig, Hannover Medical School, Hanover, Germany; ^3^Department of General, Visceral and Transplantation Surgery, Hannover Medical School, Hanover, Germany; ^4^Neuroanatomy and Cell Biology, Hannover Medical School, Hanover, Germany

**Keywords:** clostridial glycosylating toxins, human intestinal organoids, endocytosis, receptors, cell surface, *Clostridium perfringens*, fibroblasts/myofibroblast, clostridioides difficile infection

## Abstract

Toxin-producing strains of *Clostridioides difficile* and *Clostridium perfringens* cause infections of the gastrointestinal tract in humans and ruminants, with the toxins being major virulence factors, essential for the infection, and responsible for the onset of severe symptoms. *C. difficile* toxin A (TcdA) and toxin B (TcdB), and the large cytotoxin (TpeL) from *C. perfringens* are single chain bacterial protein toxins with an AB-like toxin structure. The C-terminal delivery domain mediates cell entry of the N-terminal glycosyltransferase domain by receptor-mediated endocytosis. Several cell surface proteins have been proposed to serve as toxin receptors, including chondroitin-sulfate proteoglycan 4 (CSPG4), poliovirus receptor-like 3 (PVRL3), and frizzled-1/2/7 (FZD1/2/7) for TcdB and LDL-receptor-related protein-1 (LRP1) for TpeL. The expression of the TcdB receptors was investigated in human intestinal organoids (HIOs) and in cultured cell lines. HIOs from four human donors exhibited a comparable profile of receptor expression, with PVRL3, LRP1, and FZD7 being expressed and CSPG4 and FZD2 not being expressed. In human epithelial Caco-2 cells and HT29 cells as well as in immortalized murine fibroblasts, either receptor FZD2/7, CSPG4, PVRL3, and LRP1 was expressed. The question whether the toxins take advantage of the normal turnover of their receptors (i.e., constitutive endocytosis and recycling) from the cell surface or whether the toxins activity induce the internalization of their receptors has not yet been addressed. For the analysis of receptor internalization, temperature-induced uptake of biotinylated toxin receptors into immortalized mouse embryonic fibroblasts (MEFs) and Caco-2 cells was exploited. Solely LRP1 exhibited constitutive endocytosis from the plasma membrane to the endosome, which might be abused by TpeL (and possibly TcdB as well) for cell entry. Furthermore, internalization of CSPG4, PVRL3, FZD2, and FZD7 was observed neither in MEFs nor in Caco-2 cells. FZD2/7, CSPG4, and PVRL3 did thus exhibit no constitutive recycling. The presence of TcdB and the p38 activation induced by anisomycin were not able to induce or enhance CSPG4 or PVRL3 uptake in MEFs. In conclusion, FZD2/7, CSPG4, and PVRL3 seem to serve as cell surface binding receptors rather than internalizing receptors of TcdB.

## Introduction

The large clostridial glucosylating toxins (LCGTs), are single chain bacterial protein toxins with molecular masses ranging from 191 to 308 kDa and an AB-like toxin structure. The C-terminal delivery domain mediates cell entry of the N-terminal glycosyltransferase (GT) domain by receptor-mediated endocytosis. The internalized glucosyltransferase domain associates with membrane phosphatidylserine facilitating mono-*O*-glucosylation of Rho-/Ras-GTPases. The LCGT family encompasses toxin A (TcdA) and toxin B (TcdB) from *Clostridioides difficile*, the lethal (TcsL), and the hemorrhagic toxin (TcsH) from *Clostridium sordellii*, and the *C. perfringens* large cytotoxin (TpeL). Treatment of cultured cells with TcdA and TcdB results in actin re-organization and (at higher toxin concentrations) in cell death, which correlates with a loss of colonic barrier function, massive inflammation, and the formation of pseudomembranes observed in *C. difficile*-infected patients (Genth et al., 2014; Aktories et al., 2017; Chandrasekaran and Lacy, 2017; Popoff, 2017).

Several cell surface proteins have been proposed to serve as receptors of TcdB, TcdA, and TpeL (Gerhard, 2017), including chondroitin-sulfate proteoglycan 4 (CSPG4) (Yuan et al., 2015; Gupta et al., 2017), poliovirus receptor-like 3 (PVRL3) (LaFrance et al., 2015), and Frizzled-1/2/7 (FZD1/2/7) (Tao et al., 2016; Chen et al., 2018) for TcdB, glycoprotein-96 (gp96) (Na et al., 2008), and sucrase-isomaltase (SI) (Pothoulakis et al., 1996) for TcdA, and LDL-receptor-related protein-1 (LRP1) for TpeL (Schorch et al., 2014). In addition, LRP1 has most recently been proposed to serve as a cell entry receptor for TcdB as well (TcdB receptor-2; Guo et al., 2017). The question whether the toxins take advantage of the normal turnover of their receptors (i.e., constitutive endocytosis and recycling) on the cell surface or whether the toxins activity induce the internalization of their receptors has not yet been addressed. The observations of this study suggest that LRP1 exhibits constitutive recycling from the plasma membrane to the endosome, which might be exploited by TpeL and possibly TcdB for cell entry. In contrast, FZD2/7, CSPG4 or PVRL3 did neither exhibit constitutive endocytosis nor was their endocytosis induced in the presence of TcdB.

## Results

### Expression of CSPG4, PVRL3, LRP1, and FZD2/7 in Cell Lines and Human Intestinal Organoids

**FIGURE 1:**
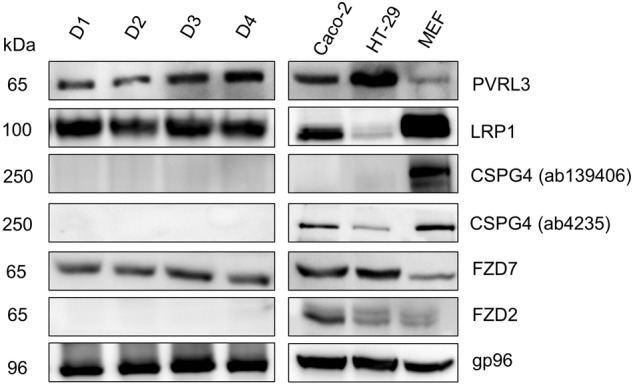
Expression of toxin receptors in human intestinal organoids (HIO) and cell lines. Human intestinal organoids from four donors (D1–4) cultured in matrigel and subconfluent human intestinal epithelial (Caco-2) cells, human colon adenocarcinoma (HT29) cells, and SV40-immortalized mouse embryonic fibroblasts (MEFs) were lysed in Laemmli sample buffer. The expression of the indicated toxin receptors was analyzed by immunoblotting with gp96 serving as loading control. Representative immune-blots are from one of three independent experiments.

Several cell lines were analyzed for expression of CSPG4 (250 kDa), PVRL3 (65 kDa), LRP1 (100 kDa), and FZD2/7 (65 kDa) (**Figure 1**). The following receptors were expressed in fetal calf serum (FCS) cultured subconfluent cells: Caco-2 cells: PVRL3, LRP1, CSPG4, FZD2/7, gp96; HT29 cells: PVRL3, FZD7, gp96, LRP1, CSPG4, and FZD2 (with the later three to minor extent); SV40-immortalized mouse embryonic fibroblasts (MEFs): LRP1, CSPG4, gp96, PVRL3, and FZD2/7 (with the latter three to minor extent). Expression of CSPG4 was analyzed exploiting the two distinct antibodies CSPG4(ab139406) and CSPG4(ab4235). Either antibody detected CSPG4 in fibroblasts, while only CSPG4(ab4235) was capable of detecting CSPG4 in Caco-2 and HT29 cells (**Figure 1**). However, these data show all TcdB receptors were expressed in any of the three cell lines tested albeit to distinct extent.

Next, expression of TcdB receptors was evaluated in human intestinal organoids (HIO) from four human donors. In all four HIOs, expression of FZD7 (65 kDa), PVRL3 (65 kDa), and LRP1 (100 kDa) but of neither CSPG4 (250 kDa) nor FZD2 was observed (**Figure 1**). The HSP90 paralog gp96, a receptor candidate for the related TcdA, was used as a loading control, as gp96 is expressed in every cell line and in every HIO tested (**Figure 1**).

### Internalization of LRP1 (Neither FZD2/7, CSPG4 Nor PVRL3) Into Fibroblasts and Caco-2 Cells

**FIGURE 2:**
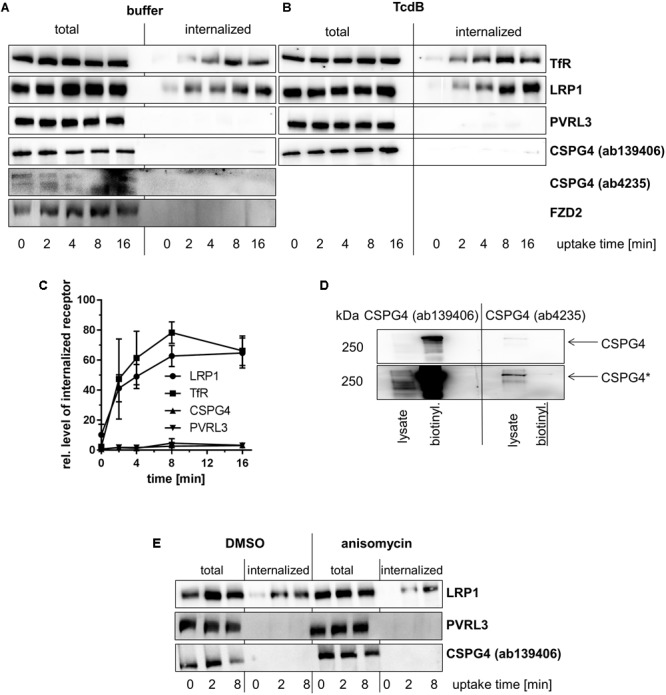
Internalization of toxin receptors. Internalization of reversibly biotinylated cell surface proteins into serum-cultured murine fibroblasts (MEFs) either left non-treated **(A)** or treated with TcdB **(B)** was induced by temperature shift to 37°C. Cells were collected at the indicated times. Cells were either left non-treated or exposed to GSH on ice to strip off biotin from still surface-exposed molecules. Biotinylated proteins were retrieved on neutravidin-agarose, eluted, and analyzed by immunoblotting. **(C)** Internalization of toxin receptors into non-treated fibroblasts was quantified by densitometry of immunoblot (*n* = 3). **(D)** Lysate from non-treated fibroblasts (lysate) and immunoprecipitate biotinylated cell surface proteins were subjected to immunoblotting and analyzed with the indicated CSPG4 antibodies. **(E)** Internalization of biotinylated cell surface proteins was further analyzed in serum-starved fibroblasts pretreated anisomycin (30 μM, **E**). Representative immunoblots are from one of three independent experiments.

**FIGURE 3:**
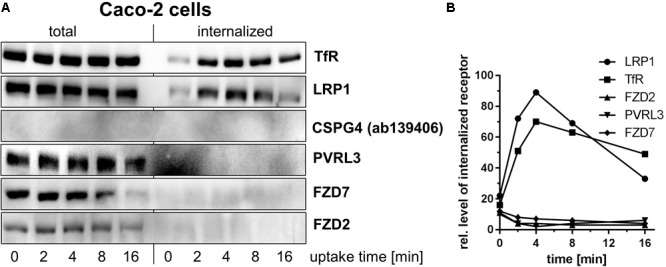
Internalization of toxin receptors into Caco-2 cells. **(A)** Internalization of reversibly biotinylated cell surface proteins into serum-cultured Caco-2 cells was induced by temperature shift to 37°C. Cells were collected at the indicated times. Cells were either left non-treated or exposed to GSH on ice to strip off biotin from still surface-exposed molecules. Biotinylated proteins were retrieved on neutravidin-agarose, eluted, and analyzed by immunoblotting. Representative immunoblots are from one of two independent experiments. **(B)** Internalization of toxin receptors was quantified by densitometry of immunoblot (*n* = 2).

Fibroblasts and Caco-2 cells were next chosen as a cell culture model for studying temperature-induced internalization of reversibly biotinylated FZD2/7, CSPG4, PVRL3, and LRP1 (Lindner, 2002; Knorr et al., 2009). Removal of all biotin, that remains surface-exposed, by treatment with cell-impermeable glutathione (GSH) on ice was exploited to differentiate between internalized and cell surface proteins. Upon cell lysis, internalized biotinylated proteins were retrieved by neutravidin-agarose matrices and their levels were analyzed by immunoblotting. The transferrin receptor (TfR) represents the prototype of a cell surface receptor that is endocytosed by clathrin-dependent endocytosis (CDE), the endocytotic pathway exploited by TcdB (Papatheodorou et al., 2010). Biotinylated TfR (i.e., TfR previously present on the cell surface) was rapidly internalized into fibroblasts (**Figures 2A–C**) and Caco-2 cells (**Figures 3A,B**), with TfR being first detected 2 min after endocytosis induction through temperature shift to 37°C. These observations served as a control for endocytosis under the chosen experimental conditions.

Internalization of PVRL3 into fibroblasts (**Figures 2A–C**) and Caco-2 cells (**Figures 3A,B**) was not observed upon endocytosis induction through temperature shift to 37°C. The latter finding suggests that PVRL3 does not serve as a primary endocytic receptor, an observation also reported for the related PVRL1 (Stiles and Krummenacher, 2010). PVRL1, however, is internalized upon binding of the HSV glycoprotein D (gD) (Stiles and Krummenacher, 2010), leading to the hypothesis that PVRL3 internalization is induced by TcdB. Internalization of PVRL3 into fibroblasts was not observed in the presence of TcdB (**Figure 2B**), suggesting that the presence of TcdB is not sufficient for inducing PVRL3 internalization.

Biotinylated CSPG4 and FZD2 were exclusively found at the plasma membrane of fibroblasts (**Figure 2A**) upon endocytosis induction through temperature shift to 37°C, suggesting that CSPG4 and FZD2 do not undergo constitutive endocytosis (**Figures 2A–C**). In Caco-2 cells, FZD2/7 was also exclusively found at the plasma membrane (**Figures 2A–C**) upon endocytosis induction, suggesting that FZD2/7 do not undergo constitutive endocytosis (**Figures 3A,B**). Remarkably, biotinylation of CSPG4 resulted in interfered immunoblot detection by CSPG4(ab4235), while biotinylated CSPG4 seems to be preferably detected by CSPG4(ab139406) (**Figure 2D**). As exclusively CSPG4(ab4235) was capable of detecting CSPG4 in Caco-2 cells (**Figure 1**), the analysis of CSPG4 internalization into Caco-2 cells was precluded. The presence of TcdB did not facilitate internalization of CSPG4 into fibroblasts, comparable to PVRL3 (**Figure 2B**). Finally, in the absence (as well as in the presence) of TcdB, LRP1 was rapidly internalized into fibroblasts with kinetics comparable to that observed for TfR (**Figures 2A–C**). Furthermore, rapid internalization of LRP1 was also observed into Caco-2 cells (**Figures 3A,B**). Out of the TcdB receptors tested, solely LRP1 exhibits constitutive recycling from the plasma membrane to the endosome, which might be exploited by TpeL and possibly TcdB for entry into fibroblast and Caco-2 cells.

### Activation of p38 MAP Kinase Does Not Facilitate the Internalization of PVRL3 and CSPG4 Into Fibroblasts

Activation of p38 MAP kinase has been shown to facilitate internalization of (non-occupied) cell surface receptors, including epidermal (EGFR) and fibroblast growth factor (FGFR) receptors (Cuadrado and Nebreda, 2010; Tomas et al., 2014; Tan et al., 2016). TcdB is well established to activate mitogen-activated protein (MAP) kinases of the p38 family (Bobo et al., 2013; Schelle et al., 2016), which leads to the hypothesis that stress-induced p38 activation facilitates internalization of PVRL3 and CSPG4. The antibiotic anisomycin, a pyrrolidine inhibitor of protein synthesis, is a potent activator of p38 MAP kinase and has been exploited as an inducer of cell surface receptor internalization in various studies (Hazzalin et al., 1998; Tan et al., 2016). Upon treatment of serum-starved MEFs, anisomycin-induced p38 activation by factor 5 ± 3 (compared with mock-treated cells), as analyzed in terms of increasing concentrations of pT180Y182–p38 MAP kinase (data not shown). Internalization of neither CSPG4 nor PVRL3 into anisomycin-treated fibroblasts was observed (**Figure 2E**), excluding p38 activation as a trigger of CSPG4 or PVRL3 internalization. In contrast, endocytosis of LRP1 into anisomycin- and control fibroblasts was similar (**Figure 2E**). In sum, endocytosis of PVRL3 and CSPG4 into fibroblasts is not observed.

## Discussion

Several cell surface receptors have been proposed for TcdB: (i) PVRL3 (also referred to as Nectin-3) belongs to the nectin family of immunoglobulin (Ig) superfamily proteins (nectin-1 to -4). PVRL1-4 are involved in cell-cell-adhesion mediated by their extracellular Ig-like ectodomains. PVRL1 and PVRL2 serve as cell entry receptors for Herpes simplex-1/2 virus (HSV-1/2) (Samanta and Almo, 2015). (ii) CSPG4 [also named nerve/glial antigen 2 (NG2)] is a single pass type I membrane glycoprotein with the core protein exhibiting a molecular mass of 250 kDa. The large ectodomain is subdivided into three structural domains, a globular domain of two laminin G-type regions (harboring the TcdB binding site), a central region of 15 repeats containing 7 Ser-Gly motifs (harboring the consensus motif SGXG for glycosaminoglycan attachment), and the membrane proximal globular D3 domain (harboring 6 of the 15 potential sites for N-linked glycosylation) (Yuan et al., 2015; Gupta et al., 2017). (iii) FZD receptors are heptahelical receptors for Wnt proteins, which bind to the extracellular cysteine-rich domain (CRD) of FZD receptors. The CRD of FZD1/2/7 has most recently been suggested to mediate cell surface binding of TcdB (Chen et al., 2018). (iv) LRP1, the cell surface receptor of the related TpeL, belongs to the low density lipoprotein (LDL) receptor family, whose members regulate lipid metabolism (Schorch et al., 2014). LRP1 further serves as a cell entry receptor of pseudomonas exoenzyme A (Kounnas et al., 1992) and of a minor-group common cold virus (Hofer et al., 1994).

Human intestinal organoids are an *in vitro* “mini-gut” model that reflects many important features of the colonic epithelium *in vivo* (Date and Sato, 2015). HIOs from four human donors exhibit a comparable expression profile for the five TcdB receptors candidates tested, with FZD7, PVRL3, and LRP1 being expressed and CSPG4 and FZD2 not being expressed (**Figure 1**). FZD7 expression seems to be required for the formation of mini-gut organoids with characteristics of the intact epithelium, as conditional deletion of FZD7 results in organoid death (Flanagan et al., 2015). A lack of CSPG4 expression has also been observed in mouse intestinal organoids (Tao et al., 2016). CSPG4 expression is found in intestinal subepithelial myofibroblasts (ISEMFs), which has led to the view that besides colonic epithelial cells ISEMFs are targeted by TcdB as well (Terada et al., 2006; Tao et al., 2016).

**FIGURE 4:**
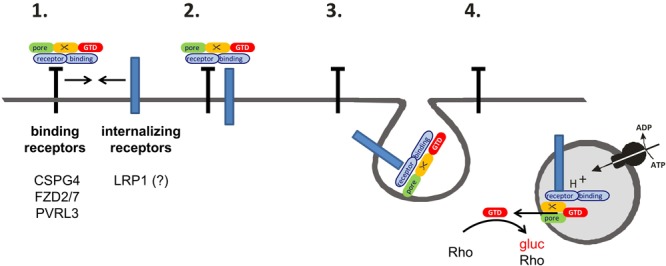
Two-receptor-model of TcdB. **(1)** TcdB binds to (non-internalizing) binding receptors at the cell surface. (Non-internalizing) binding receptors of TcdB are CSPG4, FZD2/7, and PVRL3. **(2)** The binding receptors form heterodimers with the internalizing receptors. TcdB then also binds to the internalizing receptors. The only candidate for an internalizing TcdB receptor is LRP1. **(3)** The toxin is released from the binding receptor, allowing the internalization of the TcdB-receptor complex into the early endosome. **(4)** Upon acidification, the glucosyltransferase domain (GTD) is released through a pore into the cytosol. The GTD mono-*O*-glucosylates (and thereby inactivates) Rho-GTPases.

Evaluation of the internalization of TcdB receptors into fibroblasts (**Figures 2A–C**) and Caco-2 cells (**Figures 3A,B**) revealed that LRP1 (neither of FZD2/7, CSPG4 nor PVRL3) was endocytosed to a similar extent as TfR. Comparable results were obtained in the presence of TcdB and anisomycin-induced p38 MAP kinase activation, excluding that TcdB or p38 activation triggers receptor internalization (**Figures 2C,D**). Consistently, uptake of TcdB into fibroblasts has recently been shown not to be responsive to inhibition of p38 MAP kinase (Schelle et al., 2016). These observations enforce the view that, FZD1/2/7, CSPG4, and PVRL3 are not internalizing receptors. In contrast, LRP1 exhibits constitutive recycling from the plasma membrane to the endosome, which might be exploited by TpeL (and possibly by TcdB as well) for entry into fibroblasts and Caco-2 cells. Several members of the LDL receptor family (including LRP1 and LRP5/6) have been presented to bind FZD receptors and to act as Wnt co-receptors (Zilberberg et al., 2004; Dieckmann et al., 2010). The observations of this study favor a two receptor model of the cellular uptake of TcdB (Genth and Just, 2015) (**Figure 4**): TcdB binds to FZD1/2/7, CSPG4, and PVRL3, either of which receptors exhibits (if any) a low internalization rate. Initial (low affinity) binding allows enrichment of TcdB at the surface of ISEMFs or cultured colonic epithelial cells. TcdB bound to either FZD1/2/7, CSPG4, and PVRL3 subsequently meets the receptor with a high internalization rate (possibly LRP1) that facilitates endocytosis of TcdB.

## Materials and Methods

### Ethics Statement

Surgical material from colon tissue was removed at Hannover Medical School. The study design was approved by the local ethics committee (approval number 3082-2016) and each patient has given well-informed written consent.

### Human Intestinal Organoids

Organoids were grown from surgically removed intestinal tissue using a modification of the procedure described (VanDussen et al., 2015). Briefly, the mucosa of a 0.5 cm^2^ piece of colonic tissue was lifted off the muscle layer and cut into small pieces. After washing, tissue pieces were incubated with chelating buffer [10 mM ethylenediaminetetraacetic acid (EDTA) in PBS, pH8.0] at 4°C for 90 min with shaking to remove the epithelial layer. Isolated crypts were washed twice with PBS and 500 crypts were resuspended in 50 μl growth factor reduced matrigel (Corning) and seeded into a well of pre-warmed 24-well culture dish. After solidification of matrigel for 30 min at 37°C, 1 ml of organoid growth medium (advanced DMEM/F12 supplemented with 2 mM GlutaMax, 10 mM HEPES, 100 U/ml penicillin, 100 μg/ml streptomycin, B27 supplement (1X, Gibco), 50 ng/ml recombinant EGF (Peprotech), 500 nM A83-01 (Tocris), 10 μM SB202190 (Tocris), 10 nM gastrin I (Tocris), 1 mM *N*-acetyl-L-cysteine (Sigma), and 50% supernatant of L-WRN cells (ATCC^®^ CRL-3276^TM^, containing Wnt3a, R-spondin and Noggin). 10 μM Y27623 (Tocris) was added to the medium on the day of isolation but omitted thereafter. Organoids were grown for 7 days and medium was replaced with fresh medium every other day. After 7 days, organoids from 3-wells were isolated by addition of ice-cold PBS, pooled and resuspended in 100 μl of Laemmli buffer.

### Materials

Toxin B was prepared from *C. difficile* strain VPI10463. Toxin was produced and purified yielding only one band on SDS–PAGE as previously described (Popoff, 1987; Genth et al., 2000). Anisomycin was obtained from Calbiochem, Darmstadt, Germany. Sulfo-NHS-SS-biotin and neutravidin-agarose were bought from ThermoFisher. GSH, phenylmethanesulfonyl fluoride (PMSF), E-64, and iodacetamide were purchased from Sigma. Leupeptin and pepstatin were obtained from Biomol.

### Cell Culture

SV40-immortalized MEFs, the human colon adenocarcinoma cell line HT29, and the human intestinal epithelial cell line Caco-2 were subconfluently cultured in Dulbecco’s Modified Eagle’s Medium high glucose supplemented with 10% FCS, 100 μg/ml penicillin, 100 U/ml streptomycin at 37°C and 5% CO_2_.

### Cell Surface Biotinylation and Endocytosis Assay

Cell surface biotinylation of MEFs and internalization was performed as described (Lindner, 2002; Knorr et al., 2009). Briefly, cells were detached with 7.5 mM EDTA in PBS for 10–15 min, washed and resuspended in Hanks’ balanced salt solution (HBSS). 0.5 mg/ml biotinylation reagent Sulfo-NHS-SS-Biotin (Pierce) was added for 30 min on ice before stopping the reaction with chilled culture medium supplemented with 50 mM glycine. After washes with cold HBSS, endocytosis was started by incubating the labeled cells with 37°C warm endocytosis medium (EM) for 2–16 min at 37°C. Endocytosis was stopped by adding ice-cold EM followed by GSH-stripping of remaining surface biotin. Cells were lysed in buffer (50 mM TRIS pH 7.5, 100 mM NaCl, 1% TX-100 supplemented with leupeptin, PMSF, E-64, pepstatin, and iodoacetamide). After ultracentrifugation, internalized biotinylated proteins were isolated from the supernatant with neutravidin-agarose (ThermoFisher) and subjected to immunoblotting.

### Immunoblotting

Proteins were separated using 10% polyacrylamide gels und transferred onto nitrocellulose for 2 h at 120 V, followed by blocking with 5% (w/v) non-fat dried milk for 1 h. Primary antibodies were incubated over night at 4°C with dilution according to the manufacturers’ instructions (PVRL3, 11213-1-AP, Proteintech, dilution 1:1000; TfR (H68.4), Invitrogen, dilution 1:1000; Anti-NG2/CSPG4 (ERP9195), ab139406 abcam, diution 1:1000; NG2 Antibody (#4235), cell signaling, dilution 1:1000; gp96/HSP90B1 (816803), R&D Systems, dilution 1:1000; LRP1 (EPR3724), ab92544 abcam, dilution 1:50,000; Frizzled 2, 24272-1-AP, Proteintech, dilution 1:1000; Frizzled 7, 16974-1-AP, Proteintech, dilution 1:1000) in TBST buffer (50 mM Tris–HCL, pH 7.2, 150 mM NaCl, 5 mM KCl, 0.05% (w/v) Tween 20 and subsequently for 1 h at room temperature with HRP-conjugated secondary antibody (mouse: Rockland 610-1034-121; dilution 1:5000; rabbit Rockland 611-1302; dilution 1:5000). For the chemiluminescence reaction, ECL Femto (Fisher Scientific, Schwerte, Germany) was used. The signals were detected with the INTAS Chemo Cam Imager (Intas Science Imaging Instruments GmbH, Göttingen, Germany).

## Author Contributions

HG, RL, and GG conceived the study. DS and KS performed the experiments. GG and MW supplied reagents. HG, RL, GG, and DS analyzed the data and wrote the manuscript.

## Conflict of Interest Statement

The authors declare that the research was conducted in the absence of any commercial or financial relationships that could be construed as a potential conflict of interest.
